# Reciprocal control of the stomach and intestine by viscerosensory neurons in caudal brainstem

**DOI:** 10.64898/2026.09.21.753299

**Published:** 2026-09-28

**Authors:** Naz Dundar, Ilayda Alkislar, Brooke C. Jarvie, Ho Namkung, Kathryn Xie, Mahekdeep Kaur, Queenie Li, Anagh S. Ravi, Alejandro López-Cruz, Antoinette Spina, Longhui Qiu, Truong Ly, Jun Y. Oh, Kevin Yackle, Zachary A. Knight

## Abstract

Digestion requires precisely timed transit of food through the gastrointestinal tract. Here we show that emptying of food from the stomach occurs in discrete pulses, separated by pauses that allow the proximal duodenum to clear before the next bolus arrives. By combining X-ray fluoroscopy of the gut with calcium imaging of the brainstem, we identify a population of neurons defined by expression of neuropeptide FF (NPFF) that tracks these duodenal dynamics. Stimulation of NPFF neurons induces a coordinated response that inhibits gastric emptying and accelerates intestinal transit, thereby clearing the proximal intestine for the next bolus of food. These neurons also bidirectionally control food intake through cholinergic signaling in the gut. These findings reveal a mechanism by which brainstem transforms the sensory detection of nutrients in the duodenum into the opposing control of the stomach and intestine. They also establish an experimental approach for dissecting gut-brain signaling in behaving animals.

## Full Text Availability

The license terms selected by the author(s) for this preprint version do not permit archiving in PMC. The full text is available from the preprint server.

